# Blood-brain barrier integrity and prevalence of intrathecal T helper 17.1 cells in Huntington´s disease

**DOI:** 10.1371/journal.pone.0340683

**Published:** 2026-01-12

**Authors:** Birna Ásbjörnsdóttir, Christian Sandøe Musaeus, Marie N. N. Hellem, Tua Vinther-Jensen, Patrick Ejlerskov, Esben Budtz-Jørgensen, Filippa Liliendahl Qvist, Anja Hviid Simonsen, Lena Elisabeth Hjermind, Niels Henning Skotte, Marina Rode von Essen, Finn Sellebjerg, Jørgen Erik Nielsen

**Affiliations:** 1 Neurogenetics Clinic & Research Lab, Danish Dementia Research Centre, Department of Neurology, Copenhagen University Hospital - Rigshospitalet, University of Copenhagen, Copenhagen, Denmark; 2 Section of Biostatistics, Department of Public Health, University of Copenhagen, Copenhagen, Denmark; 3 Department of Drug Design and Pharmacology, Faculty of Health and Medical Sciences, University of Copenhagen, Denmark; 4 NNF Center for Protein Research, Faculty of Health Sciences, University of Copenhagen, Copenhagen, Denmark; 5 Danish Multiple Sclerosis Centre, Department of Neurology, Copenhagen University Hospital - Rigshospitalet, Glostrup, Denmark; 6 Department of Clinical Medicine, University of Copenhagen, Copenhagen, Denmark; Beijing Chao-Yang Hospital Capital Medical University: Beijing Chaoyang Hospital, CHINA

## Abstract

**Background:**

Blood-brain barrier (BBB) involvement in the pathogenesis of Huntington´s disease (HD) is not well understood. We previously demonstrated increased prevalence of T Helper 17.1 (Th17.1) cells in the cerebrospinal fluid (CSF) of HD gene-expansion carriers (HDGECs), which might indicate a dysfunction in the BBB or the blood-CSF barrier (BCB) in HD.

**Objective:**

The aim of this exploratory study is to investigate whether the CSF/plasma albumin quotient (Q-Alb) and CSF platelet-derived growth factor-β (PDGFR-β) can be used as biomarkers for BBB/BCB integrity in HD and if there is an association between Q-Alb and the prevalence of intrathecal Th17.1 cells in HDGECs.

**Methods:**

A total of 145 HDGECs and controls were included in the Q-Alb analysis. Forty-four of these individuals underwent a second lumbar puncture after five years and were included in the analysis of changes in Q-Alb over time. CSF from 33 HDGECs and controls was analysed for Th17.1 cells and CSF from 100 HDGECs and controls was analysed for PDGFR-β.

**Results:**

No significant difference for Q-Alb was found between the pre-motor manifest HDGECs, motor manifest HDGECs, and controls (p = 0.49). We found a significant increase in Q-Alb in HDGECs over the 5-year period (p = 0.014), but when compared with controls, no significant difference was found (p = 0.32). No significant association was found between Q-Alb and the prevalence of Th17.1 cells (p = 0.97) nor Q-Alb and PDGFR-β (p = 0.89) in HDGECs.

**Conclusion:**

We found no evidence of increased BBB/BCB leakage of albumin in HDGECs compared to controls. Neither did we find signs of pericyte involvement as measured by PDGFR-β in HDGECs. These results suggest that overt BBB/BCB disruption may be limited in HDGECs. Future longitudinal studies should employ more sensitive methods like dynamic contrast-enhanced magnetic resonance imaging to evaluate region specific microleaks.

## Introduction

Huntington´s disease (HD) is an autosomal dominantly inherited neurodegenerative disease characterized by a triad of motor, cognitive, and psychiatric symptoms [1]. A CAG repeat expansion in the Huntingtin gene (*HTT*) on chromosome 4 causes HD [2]; however, the disease´s pathogenesis remains unclear. Growing evidence suggests that inflammation is a prominent characteristic of HD, which occurs both peripherally and in the central nervous system (CNS) early in the disease course [3–6].

The relationship between the peripheral and the CNS inflammation in HD is unclear, particularly whether they are independent, or whether inflammation begins in one compartment and spreads across the blood-brain barrier (BBB) or the blood-cerebrospinal fluid (CSF) barrier (BCB). The BCB regulates the exchange between blood and CSF, while BBB restricts systemic access to the CNS parenchyma [7].

We previously found more T helper 17.1 (Th17.1) cells in the CSF among HD gene-expansion carriers (HDGECs), which was accompanied by increased CSF concentrations of interleukin-17A (IL-17A) [8]. These changes were observed prior to the onset of motor symptoms, which indicate their potential role in the early stages of the disease. Migration of immune cells across BBB or BCB has previously been linked to increased BBB/BCB permeability [9,10] which includes changes in the CSF/plasma albumin quotient (Q-Alb) [11]. In addition, IL-17A has been associated with increased BBB permeability in animal studies [8,9,12]. The BCB permeability can indirectly be evaluated by measuring the Q-Alb [11,13].

Although the role of the BBB in HD is not understood, a damage to pericytes residing in the BBB, could potentially cause membrane shedding of platelet-derived growth factor receptor-β (PDGFR-β) from the pericytes [14], resulting in increased levels of PDGFR-β in the CSF [15]. This may be associated with neurodegeneration as seen in patients with Alzheimer’s disease [15,16], although this finding is not consistent across studies [17].

The aim of this hypothesis generating study was to investigate whether Q-Alb and PDGFR-β can be used as biomarkers for BBB/BCB integrity at different stages of the disease course in HDGECs. Furthermore, the aim was to examine if there is an association between Q-Alb and the prevalence of intrathecal Th17.1 cells in HDGECs.

## Materials and methods

### Participants

This study was approved by the Ethics committee of the Capital region of Denmark (H2-2011–085 and H-16047666) and written informed consent was obtained from each participant before enrolment. The participants in this study originate from two cohorts, Cohort 2013 and Cohort 2018. Participants from Cohort 2013 were recruited between January 18, 2012, and March 21, 2013, while the participants in the Cohort 2018 were recruited between June 20, 2017, and January 11, 2019. All participants were recruited from the Neurogenetics Clinic & Research Lab, Danish Dementia Research Centre, Rigshospitalet, Copenhagen, Denmark [18]. The recruitment practices were the same for the two cohorts. For details on the recruiting of participants, see Vinther-Jensen *et al.* [19] and Hellem *et al.* [20]. In short, the recruited participants were pre-motor manifest HDGECs, motor manifest HDGECs and controls. All HDGECs had CAG repeat ≥ 39 and had Unified Huntington´s Disease Rating Scale (UHDRS) - Total Motor Score (TMS) ≤ 55) [21], a Mini Mental State Examination (MMSE) score ≥ 24, and Montreal Cognitive Assessment (MoCA) score ≥ 19. Exclusion criteria included drug abuse, excessive alcohol consumption (defined as > 21 units of alcohol per week), and a native language other than Danish. Motor manifest HDGECs were defined by UHDRS-TMS > 5 [21]. The control group consisted of HD gene expansion negative individuals who were offspring of a HDGEC. All participants had been through a genetic counselling process prior to participation in this study and were aware of their own genetic status.

All participants had blood drawn and a lumbar puncture performed. CSF samples with erythrocyte count above 300 * 10^6/L were excluded due to risk of blood contamination. To our knowledge, there is no universally accepted threshold for defining blood contamination in CSF following traumatic lumbar puncture. At the Department of Clinical Biochemistry at our site, a threshold of 300 × 10⁶ erythrocytes/L has been established, and we have therefore aligned our exclusion criteria with this local standard. Moreover, we have applied the same threshold in a previous study [20].

To obtain the greatest number of participants possible when comparing Q-Alb between the three groups (pre-motor manifest HDGECs, motor manifest HDGECs, and controls), we combined all first-time plasma and CSF samples from both cohorts into a single group. In cases where a participant underwent two lumbar punctures and blood samplings, the samples from Cohort 2013 were selected. We chose this approach to ensure consistency in our selection criteria. If the CSF sample from Cohort 2013 was already excluded due to a high erythrocyte count, then the one from Cohort 2018 was chosen. We included 33 controls and 122 HDGECs, however, 9 HDGECs were excluded due to high erythrocyte count and 1 HDGEC was excluded due to missing data on CSF erythrocyte level.

Among these participants, 34 HDGECs and 10 controls underwent lumbar puncture twice as a part of Cohort 2013 and Cohort 2018 and these samples were included in the analysis of Q-Alb change over time. CSF of 36 HDGECs was analysed for prevalence of Th17.1 cells, as previously described [8], however, 3 HDGECs were excluded due to high erythrocyte count. Flow cytometric analysis was conducted exclusively on Cohort 2018 samples. CSF from 120 HDGECs and controls was analysed for PDGFR-β, and all of these originate from the Cohort 2013. Only cases with complete data for PDGFR-β and Q-Alb were included, which resulted in the exclusion of 10 cases due to missing data on PDGFR-β, 4 cases due to missing data on Q-Alb, and 6 cases due to high erythrocyte count.

### Measurements

In both cohorts, the CSF was collected in polypropylene tubes and the blood was collected in ethylenediaminetetraacetic acid (EDTA) tubes. Q-Alb analysis was performed on Cobas 8000 (Roche Diagnostics) instrument at the Department of Clinical Biochemistry at Rigshospitalet using commercial and validated kits. Plasma and CSF albumin are part of a certificated routine clinical analysis; and both were analysed immediately after sampling. Plasma and CSF albumin levels were measured by absorption and immunoturbidimetric assay, respectively. The description of Q-Alb analysis applies to both Cohort 2013 and Cohort 2018.

Flow cytometry was performed by collecting 10 mL of CSF, which was immediately centrifuged at 400 g, for 10 minutes, at 4°C, to separate the cells from the CSF. Following this, the cells were instantly immunostained and analysed by flow cytometry, and data analysis was performed. The antibody panels, gating strategy, and gating examples have been described previously [8].

PDGFR-β was assessed as part of a proteomic study using mass spectrometry (MS). Prior to analysis, the samples were stored at −80°C. Before freezing, the CSF was centrifuged at 2000 g for 10 minutes at 4°C. The samples were kept on ice until centrifugation, which was performed within one hour of sampling. The CSF samples were only thawed once, for the proteomics analysis and all samples were analysed together. Details on the MS analysis, including sample preparation, the analysis, and data processing, are available in the supplementary materials.

### Data analysis

All statistical analyses were performed using R (version 4.3.2). To compare age between motor manifest HDGECs, pre-motor manifest HDGECs, and controls, we performed a one-way ANOVA. When comparing sex, a chi-square test was performed. When comparing Q-Alb and PDGFR-β across the same three groups, we performed an ANCOVA with age as covariate, since a significant difference in age was observed between the groups. Q-Alb and PDGFR-β were logarithmically transformed to achieve normal distribution of the residuals in the model and the regression coefficients were expressed as relative changes in percent.

Since our analyses are based on pooled cohorts, we performed sensitivity analyses to examine potential clinical and demographic differences between participants included in the Cohort 2013 and the Cohort 2018, respectively. Moreover, to further assess the robustness of our results, we performed an analysis to examine a potential difference in Q-alb between the three groups among participants who underwent two lumbar punctures, using only the samples from Cohort 2018. Lastly, we added cohort (Cohort 2013 vs. 2018) as an additional covariate in our model. To investigate potential effects that may be masked in pooled analyses, we conducted exploratory subgroup analyses based on disease burden score and clinical stage. Details on these sensitivity analyses are available in supplementary materials.

When comparing changes in Q-Alb over time separately in HDGECs and controls, we performed paired Student´s t-tests. Subsequently, an unpaired Student´s t-test was used to test if a significant difference in Q-Alb change over time existed between HDGECs and controls. To compare age between the two groups, we used an unpaired Student´s t-test.

Linear regression analyses were performed to assess the relationship between Q-Alb and the prevalence of Th17.1 cells, and between Q-Alb and PDGFR-β. A p-value < 0.05 was considered statistically significant.

## Results

### Cerebrospinal fluid/plasma albumin quotient

**Fig 1:**
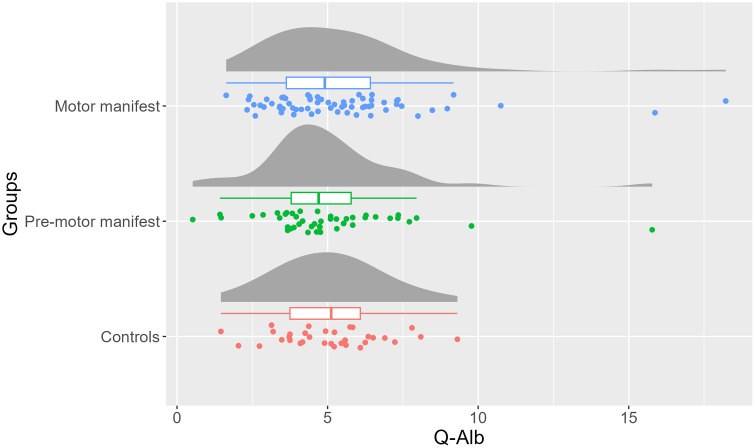
Distribution, box plot and individual data points of cerebrospinal fluid/plasma albumin quotient (Q-Alb) between motor manifest Huntington´s Disease gene-expansion carriers (HDGECs), pre-motor manifest HDGECs and controls. The boxplots display the median, the interquartile range (IQR), and the whiskers that extend to 1.5 × IQR. Group differences in Q-Alb were assessed using ANCOVA with age included as a covariate.

The demographics, UHDRS –TMS, and Q-Alb levels of the participants are shown in Table 1. A significant age difference was found between the three groups: motor manifest, pre-motor manifest, and controls (p < 0.05), but not in sex. Q-Alb was not found statistically significant between the three groups (p = 0.49; Fig 1). Q-Alb was found to be 7% (95% confidence interval (CI): −13% – 22%) lower in motor manifest HDGECs compared to controls, and 5% (95% CI: −27% − 14%) higher in premotor manifest HDGECs compared to controls when age was considered as covariate.

**Table 1:** Demographics and characteristics. HDGECs = Huntington´s disease gene-expansion carriers, N = Number of participants, SD = standard deviation, UHDRS-TMS = Unified Huntington´s Disease Rating Scale – Total Motor Score, Q-Alb = cerebrospinal fluid/plasma albumin quotient, IQR = interquartile range.

	Motor manifest HDGECs	Pre-motor manifest HDGECs	Controls
N	66	46	33
Age, mean (SD)	50.68 (11.99)	37.15 (9.95)	43.69 (13.20)
Males (N)/Females (N)	36/30	30/16	15/18
UHDRS-TMS, mean (SD)	25.82 (13.66)	1.83 (1.55)	0.51 (0.83)
Q-Alb, median (IQR)	4.91 (3.63–6.42)	4.70 (3.79–5.78)	5.12 (3.75 - 6.09)

Furthermore, no differences in Q-Alb were observed between the three groups when cohort (2013 vs. 2018) was included as an additional covariate (p = 0.96). Participants recruited in Cohort 2013 and Cohort 2018 did not differ significantly in terms of demographic or clinical characteristics. When restricting the analysis to Cohort 2018 samples among participants who underwent lumbar puncture twice, Q-Alb levels remained comparable across motor manifest, pre-motor manifest, and control groups. Additionally, no significant differences were found when both pre-motor manifest and motor manifest HDGECs were further stratified by disease burden score and clinical stage. Further details on sensitivity analyses are provided in the supplementary materials.

A total of 34 HDGECs and 10 controls had data from two lumbar punctures (see Table 2). In HDGECs, Q-Alb was found to increase by 23% (95% CI: 5% − 46%) over approximately 5 years, and the increase was found to be statistically significant (p = 0.014). In controls, Q-Alb was found to increase by 27% (95% CI: 15% − 40%) over the same period. When comparing the Q-Alb increase observed in HDGECs with controls, no significant difference was found (p = 0.32). The distribution of age and sex did not differ between HDGECs and controls.

**Table 2:** Demographic and characteristics of participants who underwent lumbar puncture twice. HDGECs = Huntington´s disease gene-expansion carriers, N = Number of participants, UHDRS-TMS = Unified Huntington´s Disease Rating Scale – Total Motor Score, SD = standard deviation, Q-Alb = cerebrospinal fluid/plasma albumin quotient, IQR = interquartile range.

	HDGECs	Controls
N	34	10
Mean age (SD) in 2013	42.65 (12.16)	42.90 (14.66)
Males (N)/Females (N)	20/14	6/4
UHDRS-TMS, Cohort 2013, mean (SD)	11.11 (12.31)	0.50 (0.97)
UHDRS-TMS, Cohort 2018, mean (SD)	23.35 (24.17)	1.4 (1.43)
Q-Alb, median (IQR), Cohort 2013	4.72 (3.79–5.93)	4.86 (3.55–5.97)
Q-Alb, median (IQR), Cohort 2018	5.76 (4.75–6.94)	5.60 (4.52–7.61)
Q-Alb difference over period of 5- years, median (IQR)	0.76 (−0.04–1.50)	1.0 (0.88–1.11)

**Fig 2:**
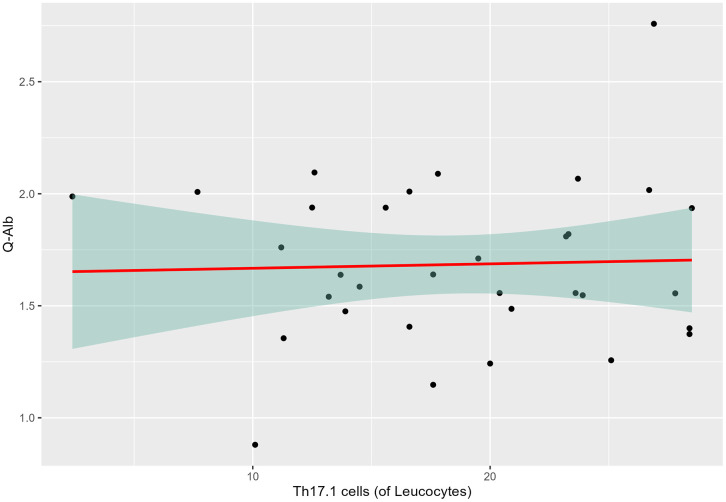
The association between the prevalence of intrathecal T helper 17.1 (Th17.1) cells and log-transformed cerebrospinal fluid/plasma albumin quotient (Q-Alb) across Huntington´s Disease Gene Expansion Carriers (HDGECs). Linear regression analysis was performed to assess the relationship between Q-Alb and the prevalence of Th17.1 cells.

Table 3 presents the demographics and characteristics of participants included in the analyses to determine if there is an association between Th17.1 cells (of leucocytes) and Q-Alb. When comparing the prevalence of intrathecal Th17.1 cells (of leucocytes) and Q-Alb levels among HDGECs, there was no significant association (p = 0.97). The slope of the line (Fig 2) indicated that when the prevalence of Th17.1 increases by 1 cell, then Q-Alb increases by 0.0036% (95%CI: −2% – 1.89%).

**Table 3:** Demographics and characteristics. HDGECs = Huntington´s disease gene-expansion carriers, N = Number of participants, SD = standard deviation, UHDRS-TMS = Unified Huntington´s Disease Rating Scale – Total Motor Score, Q-Alb = cerebrospinal fluid/plasma albumin quotient, CSF % Th17.1 cells (of leucocytes) = the prevalence of T Helper 17.1 cells out of leucocytes measured in the cerebrospinal fluid, IQR = interquartile range.

	Motor manifest HDGECs	Pre – motor manifest HDGECs
N	24	9
Age, mean (SD)	53.79 (10.36)	40.00 (8.37)
Males (N)/Females (N)	9/15	4/5
UHDRS-TMS, mean (SD)	35.04 (18.23)	1.20 (1.55)
Q-Alb, median (IQR)	5.15 (4.05–7.04)	4.74 (4.73–6.17)
CSF % Th17.1 cells (of leucocytes), mean (SD)	16.89 (6.53)	23.32 (4.76)

### Platelet-derived growth factor-β

**Fig 3:**
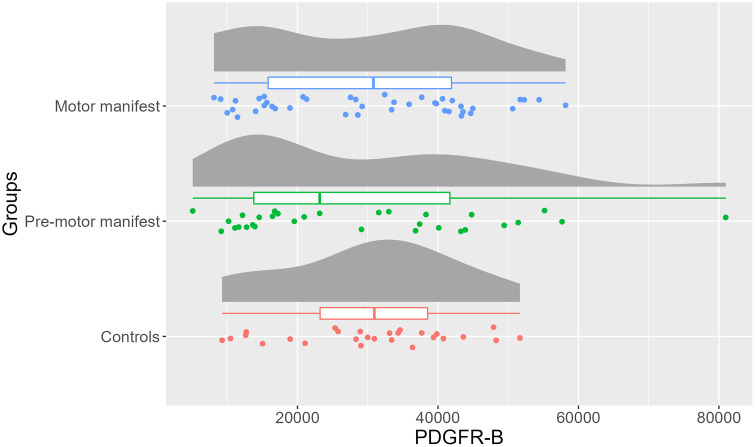
Distribution, box plot and individual data points of cerebrospinal fluid platelet-derived growth factor- β (PDGFR-B) between motor manifest Huntington´s Disease gene-expansion carriers (HDGECs), pre-motor manifest HDGECs and controls. The boxplots display the median, the interquartile range (IQR), and the whiskers that extend to 1.5 × IQR. Group differences in PDGFR-β were assessed using ANCOVA with age included as a covariate.

The CSF levels of PDGFR-β was found to be 9% lower (95% CI = −21% − 32%) in motor manifest HDGECs and 8% lower (95% CI = −26% − 33%) in pre-motor manifest HDGECs, when compared to controls and adjusting for age as a covariate (Table 4). However, these group differences did not reach statistical significance (p = 0.77; Fig 3). A significant age difference was found between the three groups (p < 0.05), but not in sex.

**Table 4:** Demographics and characteristics. HDGECs = Huntington´s disease gene-expansion carriers, N = Number of participants, SD = standard deviation, UHDRS-TMS = Unified Huntington´s Disease Rating Scale – Total Motor Score, PDGFR-β = platelet-derived growth factor receptor-β, Q-Alb = cerebrospinal fluid/plasma albumin quotient, IQR = interquartile range.

	Motor manifest HDGECs	Pre-motor manifest HDGECs	Controls
N	42	31	27
Age, mean (SD)	50.21 (12.37)	35.77 (8.54)	43.89 (13.48)
Males (N)/Females (N)	26/16	17/14	14/13
UHDRS-TMS, mean (SD)	24.17 (13.19)	1.84 (1.51)	0.41 (0.80)
PDGFR-β, median (IQR)	30,819 (15,828–41,931)	23,166 (13,786–41,692)	30,947 (23,221–38,532)
Q-Alb, median (IQR)	5.41 (4.36–6.44)	4.64 (3.67–5.83)	5.22 (3.75–5.96)

**Fig 4:**
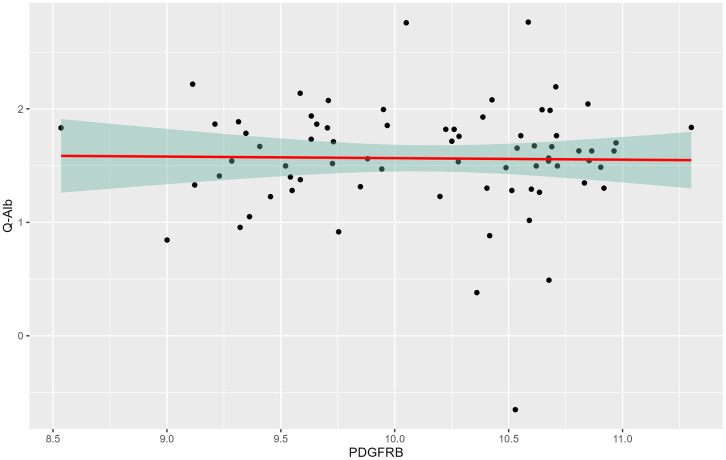
The association between log-transformed platelet-derived growth factor β (PDGFRB) in cerebrospinal fluid and log-transformed cerebrospinal fluid/plasma albumin quotient (Q-Alb) across Huntington´s disease gene expansion carriers (HDGECs). Linear regression analysis was performed to assess the relationship between Q-Alb and PDGFR- β.

No significant association was found between CSF levels of PDGFR-β and Q-Alb among HDGECs (p = 0.89). When PDGFR-β doubles, Q-Alb decreases by 1% (95% CI: −13–13%; Fig 4).

## Discussion

In the current study, we investigated Q-Alb and PDGFR-β as biomarkers for BBB/BCB integrity and whether there is an association between Q-Alb and the prevalence of intrathecal Th17.1 cells in HDGECs. The data indicate an increase in Q-Alb in both HDGECs and controls during an approximately 5-year period. This suggests increased BBB/BCB leakage of albumin with age rather than with disease progression. We found no support for an association between BBB/BCB leakage of albumin and the prevalence of Th17.1 cells, nor with levels of PDGFR-β in the CSF. Additionally, we observed no significant differences in CSF PDGFR-β across the three groups. To ensure robustness of our findings, we conducted several sensitivity analyses. These included adjusting for cohort as a covariate (2013 vs. 2018), re-running analyses using the second Q-Alb among participants with paired lumbar punctures, and further stratifying the HDGECs by disease burden score and clinical stage. None of these analyses revealed significant differences in Q-Alb levels, supporting the stability of our primary results.

Q-Alb did not differ between HDGECs and controls, consistent with a previous study investigating a small cohort of motor manifest HDGECs compared to controls [22]. However, studies have observed BBB dysfunction in HD rodent models and in brain microvasculature endothelial cells derived from human induced pluripotent stem cells generated from HDGECs [23–25]. It is therefore possible that this BBB/BCB dysfunction may be present but undetectable with Q-Alb or may affect only specific brain areas. This is supported by a post-mortem study demonstrating specific BBB impairment in putamen, with significantly decreased expression of endothelial tight junction proteins and significantly increased extravascular fibrin deposition in HDGECs [23]. Moreover, the same study using dynamic contrast enhanced (DCE) magnetic resonance imaging (MRI) showed a trend towards increased BBB permeability in the caudate nucleus and putamen, and a significant positive correlation between increased BBB permeability in the caudate nucleus and disease burden score in HDGECs [23]. This suggest that Q-Alb does not increase until later stages of the disease as previously proposed in patients with Alzheimer’s disease [26].

There may be some limitations of Q-Alb as a global marker of BBB/BCB integrity. Albumin levels in the CSF are influenced by multiple factors in the CNS including its uptake by neurons, microglia, and astrocytes, it may become proteolytically cleaved and the location of the damage of BBB may influence the entry of albumin into the CSF. CSF albumin levels may also be influenced by CSF dynamics, including flow and clearance [11]. By examining CSF biomarkers, it is not possible to identify specific brain regions with regional leakage of BBB/BCB. Detecting albumin leakage into the CSF likely requires a substantial disruption of the BCB, as albumin is a relatively large protein with a molecular weight of 66 kDa. It is also possible that evaluations of BCB cannot be used as an indicator of BBB permeability.

The lack of association between Th17.1 cell migration across the BBB/BCB and Q-Alb may be due to the possibility that Th17.1 cells migrate via transcellular routes or through tight junctions, without necessarily causing substantial disruption to the BBB or BCB, which may be necessary for increased albumin leakage across the barrier [7]. To explore this further, future studies could benefit from investigating markers, such as adhesion molecules or chemokines, that could facilitate lymphocyte entry. The chemokine CXCL10 could be a candidate, however, in our previous study [8] we observed no significant difference in CSF levels of CXCL10 between HDGECs and controls. We have previously reported that all CD4 + cells measured in the CSF in HDGECs expressed Very Late Antigen-4 (VLA-4), which is an adhesion molecule [8]. Th17.1 cells also express the adhesion molecule Mucosal Addressin Cell Adhesion Molecule (MAdCAM), which may be relevant to investigate in HDGECs [27].

To further evaluate the BBB integrity, we investigated the CSF level of PDGFR-β, which is a receptor expressed on pericytes. Pericytes play an important role in maintaining BBB integrity and PDGFR-β has been proposed as a biomarker of increased BBB permeability [14,28,29]. However, we did not find any changes in PDGFR-β when comparing the HDGECs and the control group, which is in line with the Q-Alb results. A post-mortem study found reduced pericyte coverage of blood vessels in the frontal cortex and striatum of HDGECs [30]. Hence, the reason why the PDGFR-β level is not affected is likely due to minimal damage to the BBB. As mentioned previously, it is likely that changes in the BBB are only affecting local brain regions, which may be more pronounced in the later stages of the disease.

Further investigation is needed to determine whether global BBB dysfunction as measured with Q-Alb is present in HD, how it may contribute to the pathogenesis, and whether a correlation between the BBB and neuroinflammation exists. The role of mutant huntingtin aggregates in this context remains unknown. It has been proposed that aggregates of mutant huntingtin have proinflammatory effects in immune cells [3] and a post-mortem study revealed accumulation of mutant huntingtin aggregation in brain endothelial cells, vascular smooth muscle cells, vascular basal lamina, and perivascular macrophages in HDGECs [23].

As future directions for investigating global and regional BBB/BCB disruption in HD, more sensitive methods should be used, such as DCE-MRI [31], positron emission tomography (PET) using specific tracers [32], or by analysing smaller molecules [33]. Another approach could be to investigate markers associated with structural components of the BBB, as exemplified using PDGFR-β in the present study, or molecules involved in leukocyte recruitment such as soluble intercellular adhesion molecule 1 (ICAM-1), vascular cell adhesion molecule-1 (VCAM) [34] and matrix metalloproteinase-9 (MMP-9) [35].

There are limitations in the presented study worth considering. For example, it has previously been shown that Q-Alb is affected by other factors besides age such as vascular pathology, body mass index (BMI), and degenerative spine disorders [13]. In this study we have accounted for age in our analyses when relevant, but not for other potential confounding variables. However, due to the lower age of our participants it is unlikely that they have increased vascular load or degenerative spine disorders. Nonetheless, future studies would benefit from assessing these variables. By selecting the first available Q-Alb among participants with paired samples from both cohorts, participants are younger and less affected by disease compared to samples obtained approximately five years later, which may affect our results. However, no difference in Q-Alb was found when replacing Q-Alb from Cohort 2018 instead of Cohort 2013. Lastly, the smaller number of individuals in the control group and the large variance in the measured Q-Alb and PDGFR- β may affect the statistical power of the study as illustrated by the relatively wide confidence intervals.

## Conclusion

In the presented exploratory study, we found no evidence of increased BBB/BCB leakage of albumin in HDGECs compared to controls. This is in line with previously published results on Q-Alb in HDGECs [22]. Nor did we find signs of pericyte involvement as measured by PDGFR-β in HDGECs. Since our data suggest limited loss of overt BBB/BCB integrity in HDGECs, it would be relevant to apply more sensitive methods like DCE-MRI to evaluate region specific microleaks across time.

## Supporting information

S1 File Mass spectrometry (MS) analysis and Supplementary results on sensitivity analyses of Cohort 2013 and Cohort 2018 and exploratory subgroup analyses. (DOCX)
