# Supplementary material for: Blood-brain barrier integrity and prevalence of intrathecal T helper 17.1 cells in Huntington´s disease
Source: PLoS One. 2026 Jan 12;21(1):e0340683. doi: 10.1371/journal.pone.0340683 (PMC12795374; doi:10.1371/journal.pone.0340683)
Supplement: S1 File — (DOCX) [file pone.0340683.s001.docx]

**Supplementary materials**

**Mass spectrometry (MS) analysis**

**Sample preparation:**

After sampling of the cerebrospinal fluid (CSF), the CSF samples were centrifuged at 2000 g for 10 minutes at 4 °C and then stored at -80°C. The samples were only thawed once, for the proteomics analysis.

CSF samples were mixed with PreOmics lysis buffer (PreOmics GmbH) for reduction of disulfide bridges, cysteine alkylation, and protein denaturation at 95°C for 10 min. Samples were digested using Trypsin (Sigma Aldrich) and LysC (Wako) enzyme at an order of 1:100 (w/w) for 2 hours at 37℃. The reaction was stopped using a quenching buffer (1% trifluoroacetic acid in MS-grade isopropanol). Samples were then desalted on Stagetips with two layers of SDB-RPS material (Empore), eluted in a basic buffer (1% ammonia, 80% MS grade acetonitrile in MS grade water) and vacuum centrifuged to dryness (Concentrator Plus; Eppendorf, Germany). Peptides were resuspended (0.1% trifluoroacetic acid and 5% acetonitrile in MS-grade water), and concentrations were measured by Nanodrop 2000 (Thermo Fischer Scientific, Denmark).

**MS analysis:**

Samples were measured using an EASY-nLC 1200 (Thermo FisherScientific) coupled to a Orbitrap Exploris 480 Mass Spectrometer (Thermo Fisher Scientific) via a nano-electrospray ion source (Thermo Fisher Scientific). Purified peptides were separated on 50 cm UHPLC columns with an inner diameter of 75 µm packed in-house with ReproSil-Pur C18-AQ 1.9 µm resin (Dr. Maisch GmbH). In total, 500 ng of purified peptide in buffer A* was loaded onto the column in buffer A (0.1% v/v formic acid) and eluted at 300nL/min and a temperature of 60°C by a 100-minute gradient of increasing buffer B (0.1% formic acid, 80% acetonitrile) and decreasing buffer A (0.1% formic acid). The gradient started at with 5% buffer B, gradually increasing to 23% buffer B over 82 minutes and then to 40% over 8 minutes. A steep 6-minute increase to 98% buffer B followed to then plateau at 98% for 4 minutes. To acquire MS data, the data-independent acquisition (DIA) scan mode was used for single-shot patient samples, whereas the fractionated samples of the CSF pool were acquired with a top12 data-dependent acquisition (DDA) scan mode. The mass spectrometer was operated in positive polarity mode with a 275°C ion transfer tube temperature. Both acquisition schemes were combined with the same liquid chromatography gradient. The mass spectrometer was operated by the Xcalibur software (Thermo Fisher). DDA scan settings on full MS level included an ion target value of 3×10^6^ charges in the 300–1,650 m/z range with a maximum injection time of 20 ms and a resolution of 60,000 at m/z 200. At the MS/MS level, the target value was 10^5^ charges with a maximum injection time of 60 ms and a resolution of 15,000 at m/z 200. For MS/MS events only, precursor ions with 2–5 charges that were not on the 20s dynamic exclusion list were isolated in a 1.4 m/z window. Fragmentation was performed by higher-energy C-trap dissociation (HCD) with a normalized collision energy of 27 eV. Samples were acquired in DIA mode at full MS scan resolution of 120,000, with a scan range of 300-1650 m/z, maximum injection time 50ms and automatic gain control target value 3×10^6^. Each full MS scan was followed by 33 MS/MS windows ranging across 300-1650 m/z, and the MS/MS acquisition used a resolution of 30,000 with maximum injection time 54ms and automatic gain control target value 1×10^6^.

**MS data processing:**

The MS data of the fractionated pools were used to generate a DDA-library in Spectronaut (Biognosys AG). The spectral library was used to search the MS data of the single-shot patient samples in the Spectronaut software (Biognosys AG), for final protein identification and quantitation. Protein intensities were normalized using the “Local Normalization” algorithm in Spectronaut based on a local regression model. Protein and precursor FDR of 1% were used and protein quantities were reported in samples only if the protein passed the filter (“Q-value sparse” mode data filtering). All searches were performed against the human UniProt reference proteome. Searches used carbamidomethylation as fixed modification and acetylation as variable modifications. Default settings were used for other parameters. In brief, a trypsin/P proteolytic cleavage rule was used, permitting a maximum of two missed cleavages and a peptide length of 7–52 amino acids. For PDGFR-β, we excluded samples with missing values. The coefficient of variation (CV) for PDGFR-β is 48.6% across the full dataset.

**Supplementary results on sensitivity analyses of Cohort 2013 and Cohort 2018 and exploratory subgroup analyses.**

**Demographic and clinical differences between Cohort 2013 and Cohort 2018**:

Material and methods: We performed analyses to examine potential clinical and demographic differences between participants included in the Cohort 2013 and the Cohort 2018, respectively. Participants with samples in both cohorts were excluded. To compare age, Unified Huntington´s Disease Rating Scale – Total Motor Score (UHDRS-TMS), Unified Huntington´s Disease Rating Scale – Total Functional Capacity (UHDRS-TFC) and Q-Alb between the two groups we performed unpaired Student´s t-tests. When comparing sex and the variability in participants regarding disease stage and control status between the two cohorts was assessed using a chi-squared test.

Results: We included 59 from the Cohort 2013 and 32 from the Cohort 2018. No clinical or demographic differences were found in age, sex, the variability in participants regarding disease stage and control status, UHDRS-TMS and UHDRS-TFC between the two cohorts. See supplementary table 1 for details.

|  | Cohort 2013 | Cohort 2018 | p-value |
| --- | --- | --- | --- |
| N | 59 | 32 |  |
| Age, mean (SD) | 45.34 (12.34) | 48.31 (13.85) | 0.31 |
| Males (N) /Females (N) | 35/24 | 16/16 | 0.53 |
| Motor manifest HDGECs (N)/  Pre-motor manifest HDGECs (N)/  Controls (N) | 32/14/13 | 16/10/6 | 0.73 |
| UHDRS-TMS, mean (SD) | 14.75 (16.73) | 15.56 (17.38) | 0.83 |
| UHDRS-TFC, mean (SD) | 10.80 (2.69) | 10.59 (3.08) | 0.76 |

**Supplementary table 1. Demographics and characteristics.** HDGECs = Huntington´s disease gene-expansion carriers, SD = standard deviation, N = Number of participants, UHDRS-TMS = Unified Huntington´s Disease Rating Scale – Total Motor Score, UHDRS-TFC = Unified Huntington´s Disease Rating Scale – Total Functional Capacity.

**Replacing samples from Cohort 2013 with samples from Cohort 2018 for participants with paired samples**

Material and methods: To assess the robustness of our results, we performed an analysis to examine a potential difference in Q-Alb between motor manifest Huntington´s disease gene expansion carriers (HDGECs), pre-motor manifest HDGECs and controls among participants who underwent two lumbar punctures, using only the samples from the Cohort 2018. To compare age between the three groups, we performed a one-way ANOVA. When comparing sex, a chi-square test was performed. When comparing CSF/plasma albumin quotient (Q-Alb), we performed an ANCOVA with age as covariate, since a significant difference in age was observed between the groups. Q-Alb was logarithmically transformed to achieve normal distribution of the residuals in the model.

Results: We included 22 motor manifest HDGECs, 12 pre-motor manifest HDGECs and 10 controls. Significant difference was found in age and Unified Huntington´s Disease Rating Scale – Total Motor Score (UHDRS-TMS) between the three groups, but not in sex. No significant difference was found in Q-Alb between the groups. See supplementary table 2 for details.

|  | Motor manifest HDGECs | Pre-motor manifest HDGECs | Controls | p-value |
| --- | --- | --- | --- | --- |
| N | 22 | 12 | 10 |  |
| Age, mean (SD) | 53.54 (11.25) | 38.67 (6.40) | 42.90 (14.66) | 0.002 |
| Males (N)/Females (N) | 12/10 | 8/4 | 6/4 | 0.79 |
| UHDRS-TMS, mean (SD) | 35.41 (22.04) | 1.25 (1.48) | 1.40 (1.43) | < 0.0001 |
| Q-Alb, median (IQR) | 5.17 (4.47-6.90) | 6.31 (5.60-7.65) | 5.60 (4.52-7.61) | 0.25 |

**Supplementary table 2. Demographics and characteristics of participants with paired samples only using data from Cohort 2018.** HDGECs = Huntington´s disease gene-expansion carriers, N = Number of participants, SD = standard deviation, UHDRS-TMS = Unified Huntington´s Disease Rating Scale – Total Motor Score, Q-Alb = cerebrospinal fluid/plasma albumin quotient, IQR = interquartile range.

**Exploratory subgroup analyses – Q-Alb and PDGFR-** **β**

Material and methods**:** The pre-motor manifest Huntington´s disease gene expansion carriers (HDGECs) were divided into early and late pre-motor manifest HDGECs based on their disease burden score (DBS), according to the formula (CAG – 35.5) x age in years (1). DBS ≥ 250 was the criterion for late pre-motor manifest HDGECs, while DBS < 250 was the criterion for early pre-motor manifest HDGECs. The motor manifest HDGECs were divided into moderate/advanced and early motor manifest HDGECs based on their score on Unified Huntington´s Disease Rating Scale – Total Functional Capacity (UHDRS-TFC). UHDRS-TFC 7-13 was the criterion for early motor manifest HDGECs and UHDRS-TFC < 7 was the criterion for moderate/advanced motor manifest HDGECs.

To compare age between the five groups, we performed a one-way ANOVA. When comparing sex, a chi-square test was performed. When comparing CSF/plasma albumin quotient (Q-Alb) and CSF platelet-derived growth factor- β (PDGFR- β) respectively, we performed an ANCOVA with age as covariate, since a significant difference in age was observed between the groups. Q-Alb and PDGFR-β were logarithmically transformed to achieve normal distribution of the residuals in the model.

Results for the Q-Alb: We included 11 moderate/advanced motor manifest HDGECs, 55 early motor manifest HDGECs, 22 late pre-motor manifest HDGECs, 24 early pre-motor manifest HDGECs and 33 controls. Significant difference was found in age (p = 0.002) and Unified Huntington´s Disease Rating Scale – Total Motor Score (UHDRS-TMS) (p < 0.0001) between the five groups, but not in sex (0.45). No significant difference was found in Q-Alb between the groups (p = 0.48). See supplementary table 3 for details.

Results for the PDGFR- β: We included 6 moderate/advanced motor manifest HDGECs, 36 early motor manifest HDGECs, 13 late pre-motor manifest HDGECs, 18 early pre-motor manifest HDGECs and 27 controls. Significant difference was found in age (p < 0.0001) and UHDRS-TMS (p < 0.0001) between the five groups, but not in sex (0.48). No significant difference was found in PDGFR- β between the groups (p = 0.57). See supplementary table 4 for details.

|  | **Moderate/**  **advanced**  **motor manifest HDGECs** | **Early motor manifest HDGECs** | **Late pre-motor manifest HDGECs** | **Early pre-motor manifest HDGECs** | **Controls** |
| --- | --- | --- | --- | --- | --- |
| **N** | 11 | 55 | 22 | 24 | 33 |
| Age, mean (SD) | 52.45 (10.70) | 50.33 (12.29) | 41.27 (8.99) | 33.38 (9.43) | 43.69 (13.20) |
| Males/Females | 5/6 | 31/24 | 15/7 | 15/9 | 15/18 |
| UHDRS-TMS, mean (SD) | 41.73 (12.60) | 22.64 (11.55) | 2.36 (1.68) | 1.33 (1.27) | 0.51 (0.83) |
| Q-Alb, median (IQR) | 4.67 (3.90-5.99) | 5.10 (3.57-6.46) | 4.92 (3.79-6.52) | 4.65 (3.89-5.35) | 5.12 (3.75-6.09) |

**Supplementary table 3. Demographics and characteristics of participants for subgroup analyses of Q-Alb** HDGECs = Huntington´s disease gene-expansion carriers, N = Number of participants, SD = standard deviation, UHDRS-TMS = Unified Huntington´s Disease Rating Scale – Total Motor Score, Q-Alb = cerebrospinal fluid/plasma albumin quotient, IQR = interquartile range.

|  | **Moderate/**  **advanced**  **motor manifest HDGECs** | **Early motor manifest HDGECs** | **Late pre-motor manifest HDGECs** | **Early pre-motor manifest HDGECs 56** | **Controls** |
| --- | --- | --- | --- | --- | --- |
| **N** | 6 | 36 | 13 | 18 | 27 |
| Age, mean (SD) | 51.33 (10.82) | 50.03 (12.74) | 40.15 (8.25) | 32.61 (7.43) | 43.89 (13.48) |
| Males/Females | 2/4 | 24/12 | 8/5 | 9/9 | 14/13 |
| UHDRS-TMS, mean (SD) | 38.50 (14.65) | 21.78 (11.49) | 2.23 (1.69) | 1.56 (1.34) | 0.41 (0.80) |
| PDGFR- β, median (IQR) | 35,965 (27,813-48,819) | 30,819 (15,272-41,133) | 29,960 (12,180-38,302) | 30,346 (14,088-42,479) | 30,947 (23,221-38,532) |

**Supplementary table 4. Demographics and characteristics of participants for subgroup analyses of PDGFR-** **β.** HDGECs = Huntington´s disease gene-expansion carriers, N = Number of participants, SD = standard deviation, UHDRS-TMS = Unified Huntington´s Disease Rating Scale – Total Motor Score, PDGFR-β = platelet-derived growth factor receptor-β, IQR = interquartile range.

**References:**

1. Penney JB, Vonsattel JP, MacDonald ME, Gusella JF, Myers RH. CAG repeat number governs the development rate of pathology in Huntington’s disease. Ann Neurol [Internet]. 1997 May [cited 2025 Sep 24];41(5):689–92. Available from: https://pubmed.ncbi.nlm.nih.gov/9153534/
